# Molecular Pathways in Clonal Hematopoiesis: From the Acquisition of Somatic Mutations to Transformation into Hematologic Neoplasm

**DOI:** 10.3390/life12081135

**Published:** 2022-07-28

**Authors:** Charles Gaulin, Katalin Kelemen, Cecilia Arana Yi

**Affiliations:** 1 Division of Hematology and Medical Oncology, Department of Medicine, Mayo Clinic, Phoenix, AZ 85054, USA; aranayi.cecilia@mayo.edu; 2 Division of Hematopathology, Department of Laboratory Medicine and Pathology, Mayo Clinic, Phoenix, AZ 85054, USA; kelemen.katalin@mayo.edu

**Keywords:** clonal hematopoiesis, hematopoietic stem cell, aging, hematologic neoplasms

## Abstract

Hematopoietic stem cell aging, through the acquisition of somatic mutations, gives rise to clonal hematopoiesis (CH). While a high prevalence of CH has been described in otherwise healthy older adults, CH confers an increased risk of both hematologic and non-hematologic diseases. Classification of CH into clonal hematopoiesis of indeterminate potential (CHIP) and clonal cytopenia of undetermined significance (CCUS) further describes this neoplastic myeloid precursor state and stratifies individuals at risk of developing clinically significant complications. The sequential acquisition of driver mutations, such as DNMT3A, TET2, and ASXL1, provide a selective advantage and lead to clonal expansion. Inflammation, microbiome signatures, and external selective pressures also contribute to clonal evolution. Despite significant progress in recent years, the precise molecular mechanisms driving CH transformation to hematologic neoplasms are not well defined. Further understanding of these complex mechanisms may improve risk stratification and introduce therapeutic interventions in CH. Here we discuss the genetic drivers underpinning CH, mechanisms for clonal evolution, and transformation to hematologic neoplasm.

## 1. Introduction

Hematopoietic stem cells (HSC) acquire somatic mutations with every mitotic division as we age [1,2,3]. While some mutations are passengers of minimal pathogenic consequence, others can promote cellular self-renewal, often at the cost of differentiation, and lead to clonal expansion [4]. The sequential acquisition of somatic driver mutations with oncogenic potential can shape mutant HSC clones into a neoplastic myeloid precursor state referred to broadly as clonal hematopoiesis (CH) [5,6,7,8]. Epigenetic changes, aging, inflammation, the microbiome, and the bone marrow microenvironment can all influence the landscape of CH [9,10]. In the absence of other hematologic ramifications, such as cytopenias or dysplastic hematopoiesis, this entity in stem cell biology was termed clonal hematopoiesis of indeterminate potential (CHIP) [11]. This perhaps universal marker of aging is associated with an increased risk of hematologic neoplasms, cardiovascular disease, all-cause mortality, and unexpectedly, a reduced risk of Alzheimer’s disease [12,13,14,15].

The aim of this review is to outline the role of major CH driver mutations, review the influence of other factors in CH and discuss the transformation to hematologic neoplasms, with a focus on myelodysplastic syndromes (MDS), myeloproliferative neoplasms (MPNs), and acute myeloid leukemia (AML).

## 2. Methods

We conducted a systematic review in PubMed, OVID and Medline articles using the MeSH-terms “clonal hematopoiesis”, “clonal evolution”, “aging”, and “inflammation” as title or abstract terms from January 2012 to July 2022. We reviewed the bibliography of all retrieved papers to identify relevant content in CH, CHIP, clonal evolution, and hematologic neoplasms.

## 3. Epidemiology and Definitions

The prevalence of CH increases with age, although it varies based upon the defined variant allele frequency (VAF) detection threshold [16]. Using whole-exome sequencing and a threshold VAF > 2% in subjects unselected for hematologic phenotypes, initial large population studies estimated the prevalence of CH to be at least 5% in persons older than 60 years of age, while seldom occurring in younger persons [12,14,17]. Subsequent work using techniques allowing for the detection of CH mutations with a VAF of ≥0.01% found the prevalence of CH to be nearly ubiquitous in persons older than 50 years of age, although the clinical significance of clones with such a low VAF is uncertain [18,19].

The fifth edition of the *World Health Organization (WHO) Classification of Haematolymphoid Tumours* recently defined CHIP as the presence of a somatic mutation associated with myeloid neoplasia detected in the peripheral blood or bone marrow with a VAF ≥ 2% in the absence of definitive morphologic evidence of a hematologic disorder [20]. CHIP, when an associated cytopenia is present (hemoglobin < 13 g/dL in males or <12 g/dL in females for anemia, absolute neutrophil count < 1.8 × 10^9^/L for leukopenia, and platelets < 150 × 10^9^/L for thrombocytopenia), is defined as clonal cytopenia of undetermined significance (CCUS) [20]. Composed of a mixed group in which cytopenia is present with seemingly normal bone marrow morphology without identifiable clonality, idiopathic cytopenia of undetermined significance (ICUS), although related, falls outside the scope of this review [11,21].

## 4. Acquisition of Mutations

The single cell origin hypothesis underlying cancer pathophysiology, with its hallmark stepwise acquisition of mutations conferring survival advantage, long predates our current understanding of the mechanisms driving HSC clonal evolution in CH [4,22]. Through the application of analogous principles, mutations predominantly in epigenetic regulators (DNMT3A, TET2, ASXL1, IDH1/2), splicing factors (SF3B1, SRSF2, U2AF1), DNA damage response (PPM1D, TP53), and signaling molecules (JAK2 V617F) affect HSC fitness and drive clonal expansion (Table 1) [4,9]. HSC clones with multiple drivers can then exhibit mutational cooperativity, further increasing their ability to thrive in the presence of selective pressures and establish clonal dominance [8]. Although still in early phases, our understanding of the evolutionary dynamics of CH has grown tremendously and has shed light on the early disease initiating steps of myeloid neoplasms.

### 4.1. Epigenetic Regulators

By far the most common, loss-of-function mutations in DNA methyltransferase 3 alpha (DNMT3A) are found in approximately half of individuals with CH [12,14,23]. Playing an important role in HSC development, DNMT3A encodes for a de novo DNA methyltransferase enzyme responsible for establishing DNA methylation patterns which in turn impact gene expression [24]. Physiologically, epigenetic regulation of gene expression affects cell fate decisions and ultimately defines a cell’s final differentiated state [25]. While the exact mechanisms through which DNMT3A mutations contribute to CH are not fully elucidated, in murine models, a loss of DNMT3A leads to HSC division biased towards self-renewal, causing cellular expansion at the cost differentiation [26,27]. Analysis of clonal evolution in CH and myeloid neoplasms suggest that DNMT3A mutations are likely disease-initiating, may occur at an early age, and slow in growth in older age in the context of a competitive landscape [8,28,29]. Recent studies have explored the functional consequences of specific DNMT3A variants. Huang et al. evaluated 253 disease-associated DNMT3A variants using a CRISPR screen and found that in 74% of cases these variants led to a loss of DNMT3A function [30]. Approximately half of the DNMT3A variants exhibited protein instability which in turn was associated with clonal expansion and transformation to AML [30]. Hence, not all DNMT3A variants confer the same fitness effect. For instance, highly fit DNMT3A mutations at the R882 residue in CH and AML cells are associated with DNA hypomethylation and anthracycline resistance [31]. These findings suggest that DNMT3A mutations at the R882 residue, as well as other highly fit variants, confer a significant selective advantage, are less likely to be disease-initiating, and occur later with the acquisition of other mutated genes [8,23,32,33].

Mutations in ten-eleven translocation 2 (TET2) are the second most common in CH, affecting approximately 15% of individuals [4]. Physiologically, TET proteins are ultimately involved in DNA demethylation [34]. Loss-of-function TET2 mutations in CH are associated with DNA hypermethylation in a non-random and global manner; in AML, they occur in many loci in HSCs, suggesting that these mutations are initiating events and may also result in leukemic transformation [8,35,36,37]. Data by Fabre et al. demonstrated that TET2 mutations in CH can arise across multiple age groups, exhibit a consistent growth rate over time, and eventually overtake DNMT3A as the most prevalent in older age [29]. Loss of TET2 is associated with increased cellular self-renewal and impaired differentiation. However, this effect disproportionately impacts downstream myeloid progenitors rather than long-term HSCs [38]. While their physiologic function suggests an antagonistic effect, TET2 and DNMT3A mutations can co-occur and result in synergy, as their potentials vary [4,38]. Beyond these proliferative consequences, evidence also supports a link between TET2 mutations and immune function [39]. TET2 physiologically restrains inflammatory gene expression and, accordingly, TET2 loss-of-function mutations are associated with increased levels of proinflammatory cytokines [23,40,41,42]. Pre-clinical data suggests that vitamin C treatment mimics TET2 function in TET2 deficient cells and promotes cellular differentiation [43]. As a result of these findings, clinical studies exploring the impact of vitamin C supplementation in patients with hematologic neoplasms are ongoing (NCT03682029; NCT03418038).

Addition of sex combs such as 1 (ASXL1) mutations are present in approximately 7% of patients with CH [4,44]. ASXL1 loss-of-function, dominant negative, and gain-of-function mutations have been associated with altered polycomb repressive complex function leading to histone modification and dysregulated hematopoiesis [45,46,47,48]. ASXL1 mutations in murine models with CH activate the Akt/mTOR pathway leading to HSCs proliferation and dysfunction [49]. Although not well understood, ASXL1 mutations are likely early events in CH [48,49,50]. Interestingly, ASXL1 mutations have been associated with smoking, offering a potential mechanism for the increased risk of leukemia observed in smokers, and are also linked to pesticide exposure [9,51,52,53].

Mutations in cytosolic isocitrate dehydrogenase 1 (IDH1) and its mitochondrial homolog isocitrate dehydrogenase 2 (IDH2) are far less common in CH than in AML, collectively representing <1% of mutations [4,54]. Physiologically, IDH1/2 play a key role in citrate metabolism, catalyzing the isocitrate to alpha-ketoglutarate (αKG) reaction in the Krebs cycle [55]. IDH1/2 mutations lead to the production of 2-hydroxyglutarate (2HG), which results in DNA hypermethylation, in part, through impaired αKG dependent TET2 catalytic function [54,55]. IDH1/2 and TET2 mutations are typically mutually exclusive, supporting the necessity of this downstream effect in leukemogenesis [47]. IDH1/2 mutations through these mechanisms ultimately impair cellular differentiation [55]. IDH1/2 mutations can be disease-initiating in CH, although they tend to occur later in life, and often in conjunction with DNMT3A mutations resulting in a synergistic selective advantage [8,28,56]. IDH1/2 mutations are early events in clonal evolution in MDS and AML, while they tend to appear later in MPNs, leading to leukemic transformation [57]. Reasons for their disproportionately higher implication in AML and relative absence in CH are unclear, although it could be related to differences in clonal fitness in the context of selective pressures and the surrounding microenvironment [47]. In a study incorporating subjects from the Women’s Health Initiative exploring the premalignant mutational landscape of AML, all subjects with IDH1/2 mutations eventually developed AML, highlighting the potential benefit of early intervention [58]. On a background of their efficacy in AML [59,60], phase 1 trials studying the mutant IDH1 inhibitor, ivosidenib, and the mutant IDH2 inhibitor, enasidenib, are both underway in patients with CCUS to determine their impact on hematologic parameters in this population (NCT05030441; NCT05102370).

### 4.2. Splicing Factors

Messenger RNA (mRNA) splicing plays a vital role in governing gene expression. Broadly, splicing renders pre-mRNA into its final form, through the catalysis of reactions leading to the removal of introns and retention of exons [61,62]. This complex process is orchestrated through interactions between pre-mRNA regulatory sequences, spliceosome machinery components, and specific splicing regulators [63]. The mature mRNA product is then translated into protein. Therefore, mutations resulting in alternative splicing can lead to protein products with variable functional consequences and contribute to oncogenesis through diverse pathways [64,65].

The most frequent splicing factor mutations in CH are in SF3B1, SRSF2, and U2AF1. Altogether, these make up approximately 6% of mutations observed in CH [4]. Recurrent splicing factor mutations were first observed in MDS but are also seen in AML, chronic lymphocytic leukemia, as well as other cancers [62,66,67,68]. Splicing factor mutations in CH tend to occur later in life, are associated with a rapid clonal growth rate, and a high risk of leukemic transformation [29,69]. Splicing factor and epigenetic regulator mutations often co-occur, suggesting mutational cooperativity in leukemogenesis [50,62,70]. Co-occurring IDH2 and SRSF2 mutations, for instance, result in more profound splicing alterations than either mutation alone [70]. An emerging candidate gene, ZBTB33, also links DNA methylation and mRNA splicing pathways to convey HSC selective advantage in CH and MDS, further strengthening the synergistic relationship between epigenetic regulation and post-transcriptional machinery [71]. Other oncogenic mechanisms include U2AF1 S34F related interleukin (IL) 8 upregulation, which affects bone marrow niche formation and is associated with a poor prognosis in AML [72,73]. SF3B1 and U2AF1 mutations can also cause overexpression of the highly active longer isoform of IL−1 receptor-associated kinase 4 (IRAK4), leading to activation of inflammatory signaling pathways and leukemogenesis [74,75]. A novel IRAK4 inhibitor, CA−4948, has shown promising clinical activity in individuals with MDS and AML, particularly those with splicing factor mutations, although has yet to be studied in CH [76].

### 4.3. DNA Damage Response

CH with mutated DNA damage response (DDR) related genes is of particular interest in the context of cytotoxic therapy [9,32,77]. Physiologically, the DDR maintains the integrity of the genome. When subjected to an insult, components of the DDR collectively sense DNA damage, engage repair mechanisms, and initiate various signaling pathways impacting associated cellular processes [78]. Thus, defects in these pathways can result in a diminished response to genomic instability and increased cellular proliferation.

While DDR related mutations in PPM1D and TP53 are less frequent, together making up approximately 5% of mutations in CH, clones with these mutations exhibit a selective advantage when exposed to radiotherapy, platinum agents, topoisomerase II inhibitors, and poly(adenosine diphosphate–ribose) polymerase inhibitors (PARPi) [4,9,79,80,81]. PPM1D physiologically interacts with the tumor suppressor protein, p53, ultimately leading to downregulation of p53-mediated apoptosis [82]. PPM1D mutations result in a gain-of-function truncated protein product, thereby downregulating apoptosis and promoting cellular survival [81]. Mutations leading to a loss of TP53 also provide a selective advantage through a diminished response to genomic instability [83,84]. Missense variants in the DNA binding domain of TP53 have been associated with particularly high HSC fitness [33,85]. Mutant p53 drives CH through interactions with EZH2 leading to epigenetic modulation [86]. Both PPM1D and TP53 mutations can be present at low frequencies prior to iatrogenic exposure and are enriched in therapy-related myeloid neoplasms (t-MNs) [14,81,83]. Hence, it is likely that pre-existing HSC clones harboring DDR mutations are preferentially selected when exposed to cytotoxic therapy. There has been a growing interest in better understanding the relationship between DDR mutated CH and the development of t-MNs to individualize the risk of chemotherapy [9]. In part due to their selective advantages, PPM1D and TP53 mutated t-MNs are associated with a reduced response to chemotherapy and are near-universally fatal [77,83,87,88]. PPM1D and EZH2 inhibitors may be a future approach to reduce the risk of t-MNs by preventing chemotherapy-induced selection of DDR mutated clones in those with CH [77,86].

### 4.4. Signaling Molecules

The Janus kinase–signal transducer and activator of transcription (JAK-STAT) pathway physiologically transmits signals received from extracellular polypeptides through transmembrane receptors to target gene promoters in the nucleus, thereby influencing gene expression [89]. The JAK-STAT pathway is notably essential for the signaling of several cytokines [90]. 

The Janus kinase 2 (JAK2) V617F activating mutation is present in approximately 3% of individuals with CH [91,92]. Classically associated with MPNs, the JAK2 V617F mutation confers proliferative and survival advantages in downstream hematopoietic precursors, while reducing the self-renewal capacity of individual HSCs [93,94]. JAK2 mutations can be found early in life and may hence be disease initiating. However, these clones tend to have highly variable growth rates over time [28,29,95]. JAK2-mediated expansion of progenitors is thought to act as a reservoir in which other mutations can then be acquired, and through mutational cooperativity, lead to eventual leukemic transformation [4,96].

### 4.5. Other Driver Mutations

Less commonly mutated driver genes in CH include GNAS, GNB1, CBL, N-RAS, K-RAS, RUNX1, BCOR, and RAD21, among others (Table 2) [4,97,98,99,100,101,102,103,104]. Their precise role in CH remains unclear. They are generally more common in MDS and AML, suggesting that they perhaps play a role in clonal evolution [105,106,107]. Future work is needed to better characterize the significance of these infrequent mutations in CH.

## 5. Selective Pressures and Clonal Evolution

**Figure 1:**
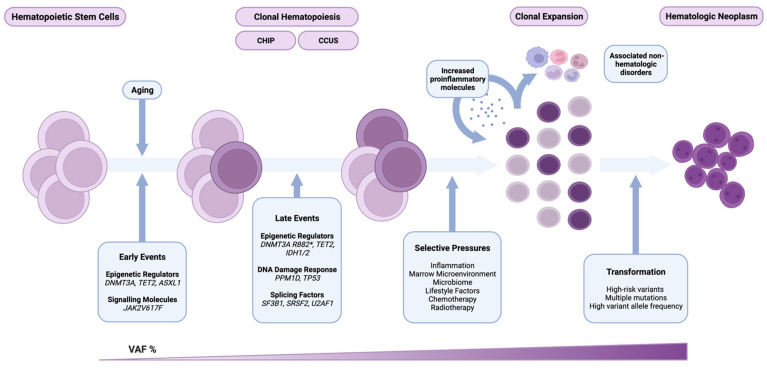
Hematopoietic stem cells (HSCs) acquire somatic mutations as we age. Mutations in DNMT3A, TET2, ASXL1, and JAK2 are early events in the development of clonal hematopoiesis (CH). Clonal hematopoiesis of indeterminant potential (CHIP), and clonal cytopenia of undetermined significance (CCUS) further describe this neoplastic myeloid precursor state. Highly fit variants in DNMT3A, such as those at the R882 residue, TET2, IDH1/2, TP53, PPM1D, and splicing factor mutations, tend to occur later and confer significant advantage in the presence of selective pressures. Inflammation, the bone marrow microenvironment, the microbiome, lifestyle factors, and cytotoxic therapy can shape CH. Mutant HSCs and leukocytes derived from them exhibit a proinflammatory phenotype reinforcing this cycle and are implicated in the pathogenesis of CH-associated non-hematologic disorders. The presence of high-risk variants, multiple co-occurring mutations, a VAF > 10%, and the presence of an associated cytopenia significantly increase the risk of malignant transformation. Created with BioRender.com.

As clones evolve, their fitness is affected by both cell-intrinsic and cell-extrinsic factors. Predominantly through positive selection, rather than genetic drift, clones with sufficient fitness harboring mutations in frequently affected genes expand and become dominant [33]. Cell-extrinsic factors, including environmental and iatrogenic exposures, shape the mutational diversity of CH. Aging, inflammation, comorbidities, lifestyle factors, chemotherapy, radiotherapy, and other factors mimicking these conditions, may impose selective pressures on HSCs. Thus, the genetic landscape of CH changes as a function of time, variant fitness effect, and mutation rate—all within the context of its surrounding environment (Figure 1).

### 5.1. Aging

Aging, through the accumulation of DNA damage, impaired mitochondrial function, and epigenetic reprogramming, leads to the functional decline of wild-type HSCs [108]. The age-related accumulation of mutant HSCs with a VAF < 2%, termed age-related clonal hematopoiesis (ARCH), is in part an adaptation to these stressors [109]. In approximately 10–40% of individuals with ARCH, mutant HSC clone size will expand to a VAF of ≥2%, consistent with CHIP [109]. Although aging is conceivably the strongest risk factor for the development of CH, somatic CH mutations can arise early in life, perhaps even in utero [110,111,112]. Germline genetic variants are associated with CH and can influence the acquisition of somatic mutations in HSCs [57,113]. For instance, asymptomatic germline RUNX1 mutation carriers have a cumulative CH risk of >80% by age 50 [114]. Age-specific mutational patterns have been found in key driver genes [28]. For instance, DNMT3A and JAK2 mutations appear throughout life, while splicing factor and IDH1/2 mutations tend to arise in individuals older than 70 years of age, suggesting that cell-extrinsic age-related factors may contribute to their acquisition [28,29,56,95]. Interestingly, studies suggest that DNMT3A mutated clones have relatively stable VAFs over the course of several years and even decelerate in older age, while TET2 mutated clones may not, highlighting the complexity of CH evolutionary dynamics [29,115,116,117].

### 5.2. Inflammation and the Bone Marrow Microenvironment

The association between inflammation and aging, termed inflammaging, is likely due to cumulative exposures to both infectious and noninfectious agents, causing a self-sustaining proinflammatory cycle [118,119]. Chronic inflammation affects the bone marrow by favoring the selection of adapted mutant HSCs and contributes to the functional decline of wild-type HSCs [119]. The bone marrow microenvironment, where HSCs reside, is increasingly recognized as a key contributor to myeloid neoplasm disease development and progression [120]. HSCs are surrounded by other cells in niches that regulate hematopoiesis by increasing inflammatory cytokines in aging [120,121,122,123]. Inflammation and cellular senescence in the mesenchymal niche induce clonal evolution and leukemic transformation in MDS and may have a similar effect in CH [124]. Moreover, leukocytes derived from mutant HSCs may then, through their proinflammatory phenotype, further contribute to this cycle [125]. DNMT3A, TET2, and JAK2 mutations are not only associated with a proinflammatory phenotype but also confer a selective advantage in this environment. DNMT3A inactivation leads to the expression of CXCL1, CXCL2, IL−6, and CCL5 in macrophage cell lines [126]. DNMT3A R878H mutated HSCs and their progenitors withstand inflammatory stressors through acquired inflammation-related cell death signaling defects [127]. TET2 deficient HSCs achieve a survival advantage through IL−6/Shp2/Stat3/Morrbid pathway overactivation [128]. JAK2 V617F mutated cells exhibit cytokine signaling overactivation leading to a proinflammatory state [129,130]. Taken together, these results suggest that CH contributes to and thrives in an inflammatory microenvironment [131].

### 5.3. The Microbiome

The microbiome, in murine models, is a partially reversible driver of HSC inflammaging, likely driven by circulating microbe-associated molecular patterns, and increased cytokine production [132,133]. The intestinal microbiota produces short-chain fatty acids which are key factors required to maintain host metabolism and immunity [134]. Lactate producing bacteria activate the secretion of stem cell factor from bone marrow mesenchymal stromal cells, which in turn activates hematopoiesis and erythropoiesis [134]. Intestinal microbiome dysbiosis is associated with oncogenesis in multiple tumor types [135]. Individuals with myeloid neoplasms have circulating microbiome dysbiosis, with significant shifts in bacterial phyla, and a reduction in α-diversity [136]. Although specific microbiome-related characteristics in CH are yet to be defined, they likely impose selective pressures on HSCs and contribute to clonal evolution.

### 5.4. Autoimmune Conditions

Autoimmune conditions may influence CH through similar inflammation-driven principles [39]. Individuals with ulcerative colitis, antineutrophil cytoplasmic antibody–associated vasculitis, and HIV appear to have higher rates of CH [137,138,139]. Interestingly, while the CH mutational distribution of individuals with vasculitis and HIV are comparable to all individuals with CH, those with ulcerative colitis tend to have PPM1D-mutated enriched CH, possibly in part due to therapy [137,138,139,140]. VEXAS (vacuoles, E1 enzyme, X-linked, autoinflammatory, somatic) syndrome, characterized by somatic mutations in UBA1 in HSCs, is associated with severe adult-onset autoinflammatory disease and hematologic neoplasms. However, perplexingly, no increased risk of CH has been observed in these individuals to date [141]. The impact of autoimmune disease on HSCs and their role in CH remains elusive and warrants further investigation.

### 5.5. Lifestyle Factors

Although less clearly defined, lifestyle factors may also affect CH. A large United Kingdom Biobank study found lower CH prevalence in individuals consuming healthier diets, with the lowest prevalence in those identifying as vegetarian [142]. Smoking is associated with greater odds of CH, and specifically, the presence of JAK2 V617F and ASLX1 mutations [9,51,91,143,144]. Alcohol consumption has also been linked to the presence of the JAK2 V617F mutation in otherwise healthy adults [91]. Furthermore, a body mass index (BMI) consistent with obesity, in comparison to a normal or overweight BMI, confers significantly higher odds of CH [143]. The precise mechanisms underlying these associations are largely unknown, although alterations in inflammation and cytokine signaling could offer a common pathway [144,145,146,147]. Collectively, these findings highlight the importance of a healthy lifestyle as a potential means to modify the natural history of CH.

## 6. Transformation to Hematologic Neoplasm

The rate of CH progression to overt hematologic neoplasia is 0.5% to 1% per year, similar to the rate observed in individuals with monoclonal gammopathy of undetermined significance [148]. This overall relatively low risk of hematologic neoplasm among all patients with CH underscores the importance of risk stratification [149]. The genes implicated, specific variants, and VAF can predict the development of an overt myeloid neoplasm [33,58,69,149]. Studies seeking to quantify the fitness advantages of key CH pathogenic variants revealed mutations in splicing factors—TP53, IDH1/2, and DNMT3A, particularly at the R882 residue—to be highly fit [9,29,33,58,69]. Fabre et al. found that in adults with CH, most clones (92.4%) remained stable even after a median follow-up of 13 years. However, mutations in DNMT3A, TP53, U2AF1, and SRSF2 had distinct growth patterns [29]. DNMT3A demonstrated early expansion, whereas TET2 clones emerged across multiple age groups. Splicing factor mutations, particularly U2AF1 and SRSF2 P95H, tended to occur later, were associated with rapid clonal growth rates, and a high risk of leukemogenesis [29]. A study by Watson et al. found that DNMT3A (R882C, R729W, R326C, R320 *, R882H, R736H, Y735C, R736C, W860R, R771 *, R598 *, P904L), SRSF2 (P95R, P95H, P95L), SF3B1 (K700E, K666N), GNB1 K57E, JAK2 V617F, and IDH2 R140Q were the most fit CH variants [33]. Individuals with one or more of the 20 most fit variants in this study were at a four-fold increased risk of developing AML when compared to those with lower-fitness variants [33]. Variants with higher frequencies are generally thought to be acquired earlier in the disease process or result in a higher fitness effect [8,33]. Correspondingly, individuals with a VAF > 10% are at a higher risk of AML development [9,58,69]. A VAF > 30%, splicing factor mutations, and co-occurring DTA (DNMT3A, TET2, ASXL1) mutations are associated with a 90% probability of myeloid malignancy progression [150]. Individuals with CCUS have perhaps the highest risk of myeloid neoplasm development, with 5- and 10-year cumulative probabilities of progression of 82% vs. 9% and 95% vs. 9%, respectively, when compared to those without identifiable clonality (ICUS) [151]. Other known AML driver mutations, such as fms-like tyrosine kinase 3 (FLT3) and nucleophosmin 1 (NPM1), are notably absent from these preleukemic HSCs and likely occur later, at the time of leukemic transformation [152,153]. Exposure to cytotoxic therapy in those with CH is associated with a ten-fold increase in the development of t-MN, largely through the selection of a highly fit dominant clone leading to progression [9,149,154,155,156]. Interestingly, however, not all t-MNs appear to arise from an antecedent CH clone, suggesting an alternative pathway where CH perhaps serves as a marker of an already strained bone marrow [149]. The association of CH with the development of lymphoid and plasma cell neoplasms, among others, is far less well described and may have different underlying pathophysiology [56]. Taken together, these data suggest that those with high-risk or multiple CH mutations, a VAF > 10%, those that have developed an associated cytopenia (CCUS), and those who have received cytotoxic therapy are at highest risk of progression to an overt myeloid neoplasm and may benefit from intervention.

## 7. Conclusions

CH is common in aging and is associated with an increased, albeit generally small, risk of hematologic neoplasm. Epigenetic changes, inflammation, the bone marrow microenvironment, microbiome signatures, and external selective pressures facilitate clonal evolution. VAF thresholds currently define CHIP and CCUS, but do not characterize other aspects of this precursor state, such as mutation type and number, both important for predicting the risk of malignant transformation. The relationship between CH and non-myeloid hematologic malignancies remains ill-defined. Although we are gaining insight into the molecular pathways of CH, we are still in the early stages of risk stratification and prevention of hematologic malignancies.

Some questions remain unanswered. What is the precise influence of aging on the bone marrow microenvironment and its role in CH? We are beginning to understand the effects of the microbiome in myeloid neoplasms. Are the microbiome signatures a consequence, a CH shaper, or initiating genotoxic agents? What are the characteristics of the microbiome in CH? Future work will be needed to characterize these findings.

The mechanisms for malignant transformation in CH are highly linked to inflammation. Is inflammation a triggering factor for CH, or is it a consequence, or is it both? What inflammatory pathways are relevant for potential preventive strategies in CH? Why certain mutations are more common in CH versus in established myeloid neoplasms is still unknown. The precise mechanisms driving malignant transformation are complex and warrant further investigation. Thus far, individuals with CH are being evaluated in multidisciplinary clinics for identification of clinical risk factors, genetic risk factors, and risk reduction strategies for myeloid neoplasms and cardiovascular disease [16,157]. Prospective analysis of patient outcomes and the development of prognostic markers for adequate CH risk stratification are needed.

## Figures and Tables

**Table 1:** Common Driver Mutations in Clonal Hematopoiesis.

Mutation Class	Prevalence	Physiologic Function	Oncogenic Mechanism
Epigenetic Regulators		Regulate gene expression through chromatin modification.	Increased cellular self-renewal and proliferation, impaired differentiation.
DNMT3A	50%		
TET2	15%		
ASXL1	7%		
IDH1/2	1%		
Splicing Factors SF3B1 SRSF2 U2AF1	6% *	Process mRNA through the removal of introns and retention of exons.	Splicing alterations affecting cellular pathways conveying increased selective advantage.
DNA Damage Response PPM1 DTP53	5% *	Maintain the integrity of the genome through repair mechanism engagement and regulation of apoptosis.	Diminished response to genomic instability and increased cellular proliferation.
Signaling Molecules JAK2 V617F	3%	Transmit extracellular signals through transmembrane receptors to target gene promoters.	Cytokine signaling overactivation, proliferative and survival advantages in downstream hematopoietic precursors.

Legend: * = collective prevalence of mutation class.

**Table 2:** Other Driver Mutations in Clonal Hematopoiesis.

Mutation Class	Prevalence	Physiologic Function
Signaling Molecules		Subunits of the heterotrimeric G-protein complex that play a role in signaling via the PI3K/AKT/mTOR and RAS/MAPK pathways. Ubiquitin ligase and multifunctional adaptor protein that regulates signal transduction. GDP–GTP-regulated binary on-off switch component of cytoplasmic signaling networks.
GNAS/GNB1	1.6%	
CBL	1.5%	
NRAS/KRAS	1.3%	
Transcription Regulation RUNX1 BCOR	6% *	Core-binding factor responsible for gene expression regulation related to hematopoiesis throughout various developmental stages of life. Corepressor of BCL−6.
Cohesin Complex RAD21 SMC1A SMC3 STAG1 STAG2	5% *	Multiprotein complex that directly interacts with DNA to maintain replication fork stability, facilitate repair of DNA damage, and maintain sister chromatid cohesion necessary for the subsequent separation of sister chromatids during anaphase.

Legend: * = collective prevalence of mutation class.

## References

[1] Busque L., Mio R., Mattioli J., Brais E., Blais N., Lalonde Y., Maragh M., Gilliland D.G. (1996). Nonrandom X-inactivation patterns in normal females: Lyonization ratios vary with age. Blood.

[2] Champion K.M., Gilbert J.G., Asimakopoulos F.A., Hinshelwood S., Green A.R. (1997). Clonal haemopoiesis in normal elderly women: Implications for the myeloproliferative disorders and myelodysplastic syndromes. Br. J. Haematol..

[3] Busque L., Patel J.P., Figueroa M.E., Vasanthakumar A., Provost S., Hamilou Z., Mollica L., Li J., Viale A., Heguy A. (2012). Recurrent somatic TET2 mutations in normal elderly individuals with clonal hematopoiesis. Nat. Genet..

[4] Challen G.A., Goodell M.A. (2020). Clonal hematopoiesis: Mechanisms driving dominance of stem cell clones. Blood.

[5] Jacobs K.B., Yeager M., Zhou W., Wacholder S., Wang Z., Rodriguez-Santiago B., Hutchinson A., Deng X., Liu C., Horner M.J. (2012). Detectable clonal mosaicism and its relationship to aging and cancer. Nat. Genet..

[6] Laurie C.C., Laurie C.A., Rice K., Doheny K.F., Zelnick L.R., McHugh C.P., Ling H., Hetrick K.N., Pugh E.W., Amos C. (2012). Detectable clonal mosaicism from birth to old age and its relationship to cancer. Nat. Genet..

[7] Welch J.S., Ley T.J., Link D.C., Miller C.A., Larson D.E., Koboldt D.C., Wartman L.D., Lamprecht T.L., Liu F., Xia J. (2012). The origin and evolution of mutations in acute myeloid leukemia. Cell.

[8] Miles L.A., Bowman R.L., Merlinsky T.R., Csete I.S., Ooi A.T., Durruthy-Durruthy R., Bowman M., Famulare C., Patel M.A., Mendez P. (2020). Single-cell mutation analysis of clonal evolution in myeloid malignancies. Nature.

[9] Bolton K.L., Ptashkin R.N., Gao T., Braunstein L., Devlin S.M., Kelly D., Patel M., Berthon A., Syed A., Yabe M. (2020). Cancer therapy shapes the fitness landscape of clonal hematopoiesis. Nat. Genet..

[10] Steensma D.P. (2018). Clinical consequences of clonal hematopoiesis of indeterminate potential. Blood Adv..

[11] Steensma D.P., Bejar R., Jaiswal S., Lindsley R.C., Sekeres M.A., Hasserjian R.P., Ebert B.L. (2015). Clonal hematopoiesis of indeterminate potential and its distinction from myelodysplastic syndromes. Blood.

[12] Jaiswal S., Fontanillas P., Flannick J., Manning A., Grauman P.V., Mar B.G., Lindsley R.C., Mermel C.H., Burtt N., Chavez A. (2014). Age-related clonal hematopoiesis associated with adverse outcomes. N. Engl. J. Med..

[13] Jaiswal S., Natarajan P., Silver A.J., Gibson C.J., Bick A.G., Shvartz E., McConkey M., Gupta N., Gabriel S., Ardissino D. (2017). Clonal Hematopoiesis and Risk of Atherosclerotic Cardiovascular Disease. N. Engl. J. Med..

[14] Genovese G., Kahler A.K., Handsaker R.E., Lindberg J., Rose S.A., Bakhoum S.F., Chambert K., Mick E., Neale B.M., Fromer M. (2014). Clonal hematopoiesis and blood-cancer risk inferred from blood DNA sequence. N. Engl. J. Med..

[15] Bouzid H., Belk J., Jan M., Qi Y., Sarnowski C., Wirth S., Ma L., Chrostek M., Ahmad H., Nachun D. (2021). Clonal Hematopoiesis is Associated with Reduced Risk of Alzheimer’s Disease. Blood.

[16] Bolton K.L., Zehir A., Ptashkin R.N., Patel M., Gupta D., Sidlow R., Papaemmanuil E., Berger M.F., Levine R.L. (2020). The Clinical Management of Clonal Hematopoiesis: Creation of a Clonal Hematopoiesis Clinic. Hematol. Oncol. Clin. N. Am..

[17] Xie M., Lu C., Wang J., McLellan M.D., Johnson K.J., Wendl M.C., McMichael J.F., Schmidt H.K., Yellapantula V., Miller C.A. (2014). Age-related mutations associated with clonal hematopoietic expansion and malignancies. Nat. Med..

[18] Young A.L., Challen G.A., Birmann B.M., Druley T.E. (2016). Clonal haematopoiesis harbouring AML-associated mutations is ubiquitous in healthy adults. Nat. Commun..

[19] Young A.L., Tong R.S., Birmann B.M., Druley T.E. (2019). Clonal hematopoiesis and risk of acute myeloid leukemia. Haematologica.

[20] Khoury J.D., Solary E., Abla O., Akkari Y., Alaggio R., Apperley J.F., Bejar R., Berti E., Busque L., Chan J.K.C. (2022). The 5th edition of the World Health Organization Classification of Haematolymphoid Tumours: Myeloid and Histiocytic/Dendritic Neoplasms. Leukemia.

[21] Valent P., Bain B.J., Bennett J.M., Wimazal F., Sperr W.R., Mufti G., Horny H.P. (2012). Idiopathic cytopenia of undetermined significance (ICUS) and idiopathic dysplasia of uncertain significance (IDUS), and their distinction from low risk MDS. Leuk. Res..

[22] Nowell P.C. (1976). The clonal evolution of tumor cell populations. Science.

[23] Buscarlet M., Provost S., Zada Y.F., Barhdadi A., Bourgoin V., Lepine G., Mollica L., Szuber N., Dube M.P., Busque L. (2017). DNMT3A and TET2 dominate clonal hematopoiesis and demonstrate benign phenotypes and different genetic predispositions. Blood.

[24] Okano M., Bell D.W., Haber D.A., Li E. (1999). DNA methyltransferases Dnmt3a and Dnmt3b are essential for de novo methylation and mammalian development. Cell.

[25] Tatapudy S., Aloisio F., Barber D., Nystul T. (2017). Cell fate decisions: Emerging roles for metabolic signals and cell morphology. EMBO Rep..

[26] Challen G.A., Sun D., Jeong M., Luo M., Jelinek J., Berg J.S., Bock C., Vasanthakumar A., Gu H., Xi Y. (2011). Dnmt3a is essential for hematopoietic stem cell differentiation. Nat. Genet..

[27] Tadokoro Y., Ema H., Okano M., Li E., Nakauchi H. (2007). De novo DNA methyltransferase is essential for self-renewal, but not for differentiation, in hematopoietic stem cells. J. Exp. Med..

[28] Acuna-Hidalgo R., Sengul H., Steehouwer M., van de Vorst M., Vermeulen S.H., Kiemeney L., Veltman J.A., Gilissen C., Hoischen A. (2017). Ultra-sensitive Sequencing Identifies High Prevalence of Clonal Hematopoiesis-Associated Mutations throughout Adult Life. Am. J. Hum. Genet..

[29] Fabre M.A., de Almeida J.G., Fiorillo E., Mitchell E., Damaskou A., Rak J., Orrù V., Marongiu M., Chapman M.S., Vijayabaskar M.S. (2022). The longitudinal dynamics and natural history of clonal haematopoiesis. Nature.

[30] Huang Y.H., Chen C.W., Sundaramurthy V., Slabicki M., Hao D., Watson C.J., Tovy A., Reyes J.M., Dakhova O., Crovetti B.R. (2022). Systematic Profiling of DNMT3A Variants Reveals Protein Instability Mediated by the DCAF8 E3 Ubiquitin Ligase Adaptor. Cancer Discov..

[31] Scheller M., Ludwig A.K., Gollner S., Rohde C., Kramer S., Stable S., Janssen M., Muller J.A., He L., Baumer N. (2021). Hotspot DNMT3A mutations in clonal hematopoiesis and acute myeloid leukemia sensitize cells to azacytidine via viral mimicry response. Nat. Cancer.

[32] Coombs C.C., Zehir A., Devlin S.M., Kishtagari A., Syed A., Jonsson P., Hyman D.M., Solit D.B., Robson M.E., Baselga J. (2017). Therapy-Related Clonal Hematopoiesis in Patients with Non-hematologic Cancers Is Common and Associated with Adverse Clinical Outcomes. Cell Stem. Cell.

[33] Watson C.J., Papula A.L., Poon G.Y.P., Wong W.H., Young A.L., Druley T.E., Fisher D.S., Blundell J.R. (2020). The evolutionary dynamics and fitness landscape of clonal hematopoiesis. Science.

[34] Kaelin W.G., McKnight S.L. (2013). Influence of metabolism on epigenetics and disease. Cell.

[35] Tulstrup M., Soerensen M., Hansen J.W., Gillberg L., Needhamsen M., Kaastrup K., Helin K., Christensen K., Weischenfeldt J., Gronbaek K. (2021). TET2 mutations are associated with hypermethylation at key regulatory enhancers in normal and malignant hematopoiesis. Nat. Commun..

[36] Abdel-Wahab O., Manshouri T., Patel J., Harris K., Yao J., Hedvat C., Heguy A., Bueso-Ramos C., Kantarjian H., Levine R.L. (2010). Genetic analysis of transforming events that convert chronic myeloproliferative neoplasms to leukemias. Cancer Res..

[37] Ortmann C.A., Kent D.G., Nangalia J., Silber Y., Wedge D.C., Grinfeld J., Baxter E.J., Massie C.E., Papaemmanuil E., Menon S. (2015). Effect of mutation order on myeloproliferative neoplasms. N. Engl. J. Med..

[38] Ostrander E.L., Kramer A.C., Mallaney C., Celik H., Koh W.K., Fairchild J., Haussler E., Zhang C.R.C., Challen G.A. (2020). Divergent Effects of Dnmt3a and Tet2 Mutations on Hematopoietic Progenitor Cell Fitness. Stem Cell Rep..

[39] Jaiswal S. (2020). Clonal hematopoiesis and nonhematologic disorders. Blood.

[40] Fuster J.J., MacLauchlan S., Zuriaga M.A., Polackal M.N., Ostriker A.C., Chakraborty R., Wu C.L., Sano S., Muralidharan S., Rius C. (2017). Clonal hematopoiesis associated with TET2 deficiency accelerates atherosclerosis development in mice. Science.

[41] Cook E.K., Izukawa T., Young S., Rosen G., Jamali M., Zhang L., Johnson D., Bain E., Hilland J., Ferrone C.K. (2019). Comorbid and inflammatory characteristics of genetic subtypes of clonal hematopoiesis. Blood Adv..

[42] Cull A.H., Snetsinger B., Buckstein R., Wells R.A., Rauh M.J. (2017). Tet2 restrains inflammatory gene expression in macrophages. Exp. Hematol..

[43] Cimmino L., Dolgalev I., Wang Y., Yoshimi A., Martin G.H., Wang J., Ng V., Xia B., Witkowski M.T., Mitchell-Flack M. (2017). Restoration of TET2 Function Blocks Aberrant Self-Renewal and Leukemia Progression. Cell.

[44] Shlush L.I. (2018). Age-related clonal hematopoiesis. Blood.

[45] Abdel-Wahab O., Adli M., LaFave L.M., Gao J., Hricik T., Shih A.H., Pandey S., Patel J.P., Chung Y.R., Koche R. (2012). ASXL1 mutations promote myeloid transformation through loss of PRC2-mediated gene repression. Cancer Cell.

[46] Abdel-Wahab O., Gao J., Adli M., Dey A., Trimarchi T., Chung Y.R., Kuscu C., Hricik T., Ndiaye-Lobry D., Lafave L.M. (2013). Deletion of Asxl1 results in myelodysplasia and severe developmental defects in vivo. J. Exp. Med..

[47] Bowman R.L., Busque L., Levine R.L. (2018). Clonal Hematopoiesis and Evolution to Hematopoietic Malignancies. Cell Stem Cell.

[48] Fujino T., Kitamura T. (2020). ASXL1 mutation in clonal hematopoiesis. Exp. Hematol..

[49] Fujino T., Goyama S., Sugiura Y., Inoue D., Asada S., Yamasaki S., Matsumoto A., Yamaguchi K., Isobe Y., Tsuchiya A. (2021). Mutant ASXL1 induces age-related expansion of phenotypic hematopoietic stem cells through activation of Akt/mTOR pathway. Nat. Commun..

[50] Rodrigues C.P., Shvedunova M., Akhtar A. (2020). Epigenetic Regulators as the Gatekeepers of Hematopoiesis. Trends Genet..

[51] Dawoud A.A.Z., Tapper W.J., Cross N.C.P. (2020). Clonal myelopoiesis in the UK Biobank cohort: ASXL1 mutations are strongly associated with smoking. Leukemia.

[52] Sandler D.P., Shore D.L., Anderson J.R., Davey F.R., Arthur D., Mayer R.J., Silver R.T., Weiss R.B., Moore J.O., Schiffer C.A. (1993). Cigarette smoking and risk of acute leukemia: Associations with morphology and cytogenetic abnormalities in bone marrow. J. Natl. Cancer Inst..

[53] Van Zeventer I.A., Salzbrunn J.B., de Graaf A.O., van der Reijden B.A., Boezen H.M., Vonk J.M., van der Harst P., Schuringa J.J., Jansen J.H., Huls G. (2021). Prevalence, predictors, and outcomes of clonal hematopoiesis in individuals aged ≥ 80 years. Blood Adv..

[54] Figueroa M.E., Abdel-Wahab O., Lu C., Ward P.S., Patel J., Shih A., Li Y., Bhagwat N., Vasanthakumar A., Fernandez H.F. (2010). Leukemic IDH1 and IDH2 mutations result in a hypermethylation phenotype, disrupt TET2 function, and impair hematopoietic differentiation. Cancer Cell.

[55] Ward P.S., Patel J., Wise D.R., Abdel-Wahab O., Bennett B.D., Coller H.A., Cross J.R., Fantin V.R., Hedvat C.V., Perl A.E. (2010). The common feature of leukemia-associated IDH1 and IDH2 mutations is a neomorphic enzyme activity converting alpha-ketoglutarate to 2-hydroxyglutarate. Cancer Cell.

[56] Stengel A., Baer C., Walter W., Meggendorfer M., Kern W., Haferlach T., Haferlach C. (2021). Mutational patterns and their correlation to CHIP-related mutations and age in hematological malignancies. Blood Adv..

[57] Valent P., Kern W., Hoermann G., Milosevic Feenstra J.D., Sotlar K., Pfeilstocker M., Germing U., Sperr W.R., Reiter A., Wolf D. (2019). Clonal Hematopoiesis with Oncogenic Potential (CHOP): Separation from CHIP and Roads to AML. Int. J. Mol. Sci..

[58] Desai P., Mencia-Trinchant N., Savenkov O., Simon M.S., Cheang G., Lee S., Samuel M., Ritchie E.K., Guzman M.L., Ballman K.V. (2018). Somatic mutations precede acute myeloid leukemia years before diagnosis. Nat. Med..

[59] DiNardo C.D., Stein E.M., de Botton S., Roboz G.J., Altman J.K., Mims A.S., Swords R., Collins R.H., Mannis G.N., Pollyea D.A. (2018). Durable Remissions with Ivosidenib in IDH1-Mutated Relapsed or Refractory AML. N. Engl. J. Med..

[60] Stein E.M., DiNardo C.D., Pollyea D.A., Fathi A.T., Roboz G.J., Altman J.K., Stone R.M., DeAngelo D.J., Levine R.L., Flinn I.W. (2017). Enasidenib in mutant IDH2 relapsed or refractory acute myeloid leukemia. Blood.

[61] Darnell J.E. (2013). Reflections on the history of pre-mRNA processing and highlights of current knowledge: A unified picture. RNA.

[62] Saez B., Walter M.J., Graubert T.A. (2017). Splicing factor gene mutations in hematologic malignancies. Blood.

[63] Coltri P.P., Dos Santos M.G.P., da Silva G.H.G. (2019). Splicing and cancer: Challenges and opportunities. Wiley Interdiscip. Rev. RNA.

[64] Rahman M.A., Krainer A.R., Abdel-Wahab O. (2020). SnapShot: Splicing Alterations in Cancer. Cell.

[65] Dvinge H., Kim E., Abdel-Wahab O., Bradley R.K. (2016). RNA splicing factors as oncoproteins and tumour suppressors. Nat. Rev. Cancer.

[66] Graubert T.A., Shen D., Ding L., Okeyo-Owuor T., Lunn C.L., Shao J., Krysiak K., Harris C.C., Koboldt D.C., Larson D.E. (2011). Recurrent mutations in the U2AF1 splicing factor in myelodysplastic syndromes. Nat. Genet..

[67] Yoshida K., Sanada M., Shiraishi Y., Nowak D., Nagata Y., Yamamoto R., Sato Y., Sato-Otsubo A., Kon A., Nagasaki M. (2011). Frequent pathway mutations of splicing machinery in myelodysplasia. Nature.

[68] Papaemmanuil E., Cazzola M., Boultwood J., Malcovati L., Vyas P., Bowen D., Pellagatti A., Wainscoat J.S., Hellstrom-Lindberg E., Gambacorti-Passerini C. (2011). Somatic SF3B1 mutation in myelodysplasia with ring sideroblasts. N. Engl. J. Med..

[69] Abelson S., Collord G., Ng S.W.K., Weissbrod O., Mendelson Cohen N., Niemeyer E., Barda N., Zuzarte P.C., Heisler L., Sundaravadanam Y. (2018). Prediction of acute myeloid leukaemia risk in healthy individuals. Nature.

[70] Yoshimi A., Lin K.T., Wiseman D.H., Rahman M.A., Pastore A., Wang B., Lee S.C., Micol J.B., Zhang X.J., de Botton S. (2019). Coordinated alterations in RNA splicing and epigenetic regulation drive leukaemogenesis. Nature.

[71] Beauchamp E.M., Leventhal M., Bernard E., Hoppe E.R., Todisco G., Creignou M., Galli A., Castellano C.A., McConkey M., Tarun A. (2021). ZBTB33 is mutated in clonal hematopoiesis and myelodysplastic syndromes and impacts RNA splicing. Blood Cancer Discov..

[72] Kuett A., Rieger C., Perathoner D., Herold T., Wagner M., Sironi S., Sotlar K., Horny H.P., Deniffel C., Drolle H. (2015). IL-8 as mediator in the microenvironment-leukaemia network in acute myeloid leukaemia. Sci. Rep..

[73] Chen S., Benbarche S., Abdel-Wahab O. (2021). Splicing factor mutations in hematologic malignancies. Blood.

[74] Choudhary G.S., Smith M.A., Pellagatti A., Bhagat T.D., Gordon S., Pandey S., Shah N., Aluri S., Booher R.N., Ramachandra M. (2019). SF3B1 Mutations Induce Oncogenic IRAK4 Isoforms and Activate Targetable Innate Immune Pathways in MDS and AML. Blood.

[75] Smith M.A., Choudhary G.S., Pellagatti A., Choi K., Bolanos L.C., Bhagat T.D., Gordon-Mitchell S., Von Ahrens D., Pradhan K., Steeples V. (2019). U2AF1 mutations induce oncogenic IRAK4 isoforms and activate innate immune pathways in myeloid malignancies. Nat. Cell Biol..

[76] Garcia-Manero G., Tarantolo S., Verma A., Dugan J., Winer E., Giagounidis A. A Phase 1, dose escalation trial with novel oral irak4 inhibitor ca-4948 in patients with acute myelogenous leukemia or myelodysplastic syndrome–interim report. Proceedings of the EHA Annual Meeting.

[77] Kahn J.D., Miller P.G., Silver A.J., Sellar R.S., Bhatt S., Gibson C., McConkey M., Adams D., Mar B., Mertins P. (2018). PPM1D-truncating mutations confer resistance to chemotherapy and sensitivity to PPM1D inhibition in hematopoietic cells. Blood.

[78] Jackson S.P., Bartek J. (2009). The DNA-damage response in human biology and disease. Nature.

[79] Kwan T.T., Oza A.M., Tinker A.V., Ray-Coquard I., Oaknin A., Aghajanian C., Lorusso D., Colombo N., Dean A., Weberpals J. (2021). Preexisting TP53-Variant Clonal Hematopoiesis and Risk of Secondary Myeloid Neoplasms in Patients with High-grade Ovarian Cancer Treated with Rucaparib. JAMA Oncol..

[80] Bolton K.L., Moukarzel L.A., Ptashkin R., Gao T., Patel M., Caltabellotta N., Braunstein L.Z., Aghajanian C., Hyman D.M., Berger M.F. (2020). The impact of poly ADP ribose polymerase (PARP) inhibitors on clonal hematopoiesis. J. Clin. Oncol..

[81] Hsu J.I., Dayaram T., Tovy A., De Braekeleer E., Jeong M., Wang F., Zhang J., Heffernan T.P., Gera S., Kovacs J.J. (2018). PPM1D Mutations Drive Clonal Hematopoiesis in Response to Cytotoxic Chemotherapy. Cell Stem Cell.

[82] Dudgeon C., Shreeram S., Tanoue K., Mazur S.J., Sayadi A., Robinson R.C., Appella E., Bulavin D.V. (2013). Genetic variants and mutations of PPM1D control the response to DNA damage. Cell Cycle.

[83] Wong T.N., Ramsingh G., Young A.L., Miller C.A., Touma W., Welch J.S., Lamprecht T.L., Shen D., Hundal J., Fulton R.S. (2015). Role of TP53 mutations in the origin and evolution of therapy-related acute myeloid leukaemia. Nature.

[84] Chen X., Ko L.J., Jayaraman L., Prives C. (1996). p53 levels, functional domains, and DNA damage determine the extent of the apoptotic response of tumor cells. Genes Dev..

[85] Boettcher S., Miller P.G., Sharma R., McConkey M., Leventhal M., Krivtsov A.V., Giacomelli A.O., Wong W., Kim J., Chao S. (2019). A dominant-negative effect drives selection of TP53 missense mutations in myeloid malignancies. Science.

[86] Chen S., Wang Q., Yu H., Capitano M.L., Vemula S., Nabinger S.C., Gao R., Yao C., Kobayashi M., Geng Z. (2019). Mutant p53 drives clonal hematopoiesis through modulating epigenetic pathway. Nat. Commun..

[87] Kayser S., Dohner K., Krauter J., Kohne C.H., Horst H.A., Held G., von Lilienfeld-Toal M., Wilhelm S., Kundgen A., Gotze K. (2011). The impact of therapy-related acute myeloid leukemia (AML) on outcome in 2853 adult patients with newly diagnosed AML. Blood.

[88] Fianchi L., Pagano L., Piciocchi A., Candoni A., Gaidano G., Breccia M., Criscuolo M., Specchia G., Maria Pogliani E., Maurillo L. (2015). Characteristics and outcome of therapy-related myeloid neoplasms: Report from the Italian network on secondary leukemias. Am. J. Hematol..

[89] Aaronson D.S., Horvath C.M. (2002). A road map for those who don’t know JAK-STAT. Science.

[90] Parganas E., Wang D., Stravopodis D., Topham D.J., Marine J.C., Teglund S., Vanin E.F., Bodner S., Colamonici O.R., van Deursen J.M. (1998). Jak2 is essential for signaling through a variety of cytokine receptors. Cell.

[91] Cordua S., Kjaer L., Skov V., Pallisgaard N., Hasselbalch H.C., Ellervik C. (2019). Prevalence and phenotypes of JAK2 V617F and calreticulin mutations in a Danish general population. Blood.

[92] Hinds D.A., Barnholt K.E., Mesa R.A., Kiefer A.K., Do C.B., Eriksson N., Mountain J.L., Francke U., Tung J.Y., Nguyen H.M. (2016). Germ line variants predispose to both JAK2 V617F clonal hematopoiesis and myeloproliferative neoplasms. Blood.

[93] Levine R.L., Wadleigh M., Cools J., Ebert B.L., Wernig G., Huntly B.J., Boggon T.J., Wlodarska I., Clark J.J., Moore S. (2005). Activating mutation in the tyrosine kinase JAK2 in polycythemia vera, essential thrombocythemia, and myeloid metaplasia with myelofibrosis. Cancer Cell.

[94] Kralovics R., Passamonti F., Buser A.S., Teo S.S., Tiedt R., Passweg J.R., Tichelli A., Cazzola M., Skoda R.C. (2005). A gain-of-function mutation of JAK2 in myeloproliferative disorders. N. Engl. J. Med..

[95] McKerrell T., Park N., Moreno T., Grove C.S., Ponstingl H., Stephens J., Crawley C., Craig J., Scott M.A., Hodkinson C. (2015). Leukemia-associated somatic mutations drive distinct patterns of age-related clonal hemopoiesis. Cell Rep..

[96] Kent D.G., Li J., Tanna H., Fink J., Kirschner K., Pask D.C., Silber Y., Hamilton T.L., Sneade R., Simons B.D. (2013). Self-renewal of single mouse hematopoietic stem cells is reduced by JAK2V617F without compromising progenitor cell expansion. PLoS Biol..

[97] Asada S., Kitamura T. (2021). Clonal hematopoiesis and associated diseases: A review of recent findings. Cancer Sci..

[98] Vedula R.S., Luskin M.R., Copson K., Kumari P., Charles A., Kim A.S., Morgan E.A., Abel G.A., Stone R.M., Lane A.A. (2018). Clinical Characteristics of GNB1 and GNAS Mutations in an Unselected Cohort of 6343 Patients with Hematologic Abnormalities. Blood.

[99] Schmidt M.H.H., Dikic I. (2005). The Cbl interactome and its functions. Nat. Rev. Mol. Cell Biol..

[100] Hobbs G.A., Der C.J., Rossman K.L. (2016). RAS isoforms and mutations in cancer at a glance. J. Cell Sci..

[101] Sood R., Kamikubo Y., Liu P. (2017). Role of RUNX1 in hematological malignancies. Blood.

[102] de Bruijn M., Dzierzak E. (2017). Runx transcription factors in the development and function of the definitive hematopoietic system. Blood.

[103] Huynh K.D., Fischle W., Verdin E., Bardwell V.J. (2000). BCoR, a novel corepressor involved in BCL-6 repression. Genes Dev..

[104] Sasca D., Yun H., Giotopoulos G., Szybinski J., Evan T., Wilson N.K., Gerstung M., Gallipoli P., Green A.R., Hills R. (2019). Cohesin-dependent regulation of gene expression during differentiation is lost in cohesin-mutated myeloid malignancies. Blood.

[105] Papaemmanuil E., Gerstung M., Malcovati L., Tauro S., Gundem G., Van Loo P., Yoon C.J., Ellis P., Wedge D.C., Pellagatti A. (2013). Clinical and biological implications of driver mutations in myelodysplastic syndromes. Blood.

[106] Walter M.J., Shen D., Shao J., Ding L., White B.S., Kandoth C., Miller C.A., Niu B., McLellan M.D., Dees N.D. (2013). Clonal diversity of recurrently mutated genes in myelodysplastic syndromes. Leukemia.

[107] Haferlach T., Nagata Y., Grossmann V., Okuno Y., Bacher U., Nagae G., Schnittger S., Sanada M., Kon A., Alpermann T. (2014). Landscape of genetic lesions in 944 patients with myelodysplastic syndromes. Leukemia.

[108] de Haan G., Lazare S.S. (2018). Aging of hematopoietic stem cells. Blood.

[109] Busque L., Buscarlet M., Mollica L., Levine R.L. (2018). Concise Review: Age-Related Clonal Hematopoiesis: Stem Cells Tempting the Devil. Stem Cells.

[110] Fabre M.A., McKerrell T., Zwiebel M., Vijayabaskar M.S., Park N., Wells P.M., Rad R., Deloukas P., Small K., Steves C.J. (2020). Concordance for clonal hematopoiesis is limited in elderly twins. Blood.

[111] Williams N., Lee J., Moore L., Baxter J.E., Hewinson J., Dawson K.J., Menzies A., Godfrey A.L., Green A.R., Campbell P.J. (2020). Driver Mutation Acquisition in Utero and Childhood Followed by Lifelong Clonal Evolution Underlie Myeloproliferative Neoplasms. Blood.

[112] Van Egeren D., Escabi J., Nguyen M., Liu S., Reilly C.R., Patel S., Kamaz B., Kalyva M., DeAngelo D.J., Galinsky I. (2021). Reconstructing the Lineage Histories and Differentiation Trajectories of Individual Cancer Cells in Myeloproliferative Neoplasms. Cell Stem Cell.

[113] Bick A.G., Weinstock J.S., Nandakumar S.K., Fulco C.P., Bao E.L., Zekavat S.M., Szeto M.D., Liao X., Leventhal M.J., Nasser J. (2020). Inherited causes of clonal haematopoiesis in 97,691 whole genomes. Nature.

[114] Churpek J.E., Pyrtel K., Kanchi K.L., Shao J., Koboldt D., Miller C.A., Shen D., Fulton R., O’Laughlin M., Fronick C. (2015). Genomic analysis of germ line and somatic variants in familial myelodysplasia/acute myeloid leukemia. Blood.

[115] Kusne Y., Lasho T., Mangaonkar A., Tefferi A., Gangat N., Finke C., Binder M., Chia N., Patnaik M.M. (2021). Remarkable stability in clonal hematopoiesis involving leukemia-driver genes in patients without underlying myeloid neoplasms. Am. J. Hematol..

[116] Kusne Y., Xie Z., Patnaik M.M. (2022). Clonal hematopoiesis: Molecular and clinical implications. Leuk. Res..

[117] Arends C.M., Galan-Sousa J., Hoyer K., Chan W., Jager M., Yoshida K., Seemann R., Noerenberg D., Waldhueter N., Fleischer-Notter H. (2018). Hematopoietic lineage distribution and evolutionary dynamics of clonal hematopoiesis. Leukemia.

[118] Vasto S., Candore G., Balistreri C.R., Caruso M., Colonna-Romano G., Grimaldi M.P., Listi F., Nuzzo D., Lio D., Caruso C. (2007). Inflammatory networks in ageing, age-related diseases and longevity. Mech. Ageing Dev..

[119] Caiado F., Pietras E.M., Manz M.G. (2021). Inflammation as a regulator of hematopoietic stem cell function in disease, aging, and clonal selection. J. Exp. Med..

[120] Mian S.A., Bonnet D. (2021). Nature or Nurture? Role of the Bone Marrow Microenvironment in the Genesis and Maintenance of Myelodysplastic Syndromes. Cancers.

[121] Vas V., Senger K., Dorr K., Niebel A., Geiger H. (2012). Aging of the microenvironment influences clonality in hematopoiesis. PLoS ONE.

[122] Mosteo L., Storer J., Batta K., Searle E.J., Duarte D., Wiseman D.H. (2021). The Dynamic Interface between the Bone Marrow Vascular Niche and Hematopoietic Stem Cells in Myeloid Malignancy. Front Cell Dev. Biol..

[123] Trowbridge J.J., Starczynowski D.T. (2021). Innate immune pathways and inflammation in hematopoietic aging, clonal hematopoiesis, and MDS. J. Exp. Med..

[124] Pronk E., Raaijmakers M. (2019). The mesenchymal niche in MDS. Blood.

[125] Cook E.K., Luo M., Rauh M.J. (2020). Clonal hematopoiesis and inflammation: Partners in leukemogenesis and comorbidity. Exp. Hematol..

[126] Sano S., Oshima K., Wang Y., Katanasaka Y., Sano M., Walsh K. (2018). CRISPR-Mediated Gene Editing to Assess the Roles of Tet2 and Dnmt3a in Clonal Hematopoiesis and Cardiovascular Disease. Circ. Res..

[127] Liao M., Chen R., Yang Y., He H., Xu L., Jiang Y., Guo Z., He W., Jiang H., Wang J. (2022). Aging-elevated inflammation promotes DNMT3A R878H-driven clonal hematopoiesis. Acta Pharm. Sin. B.

[128] Cai Z., Kotzin J.J., Ramdas B., Chen S., Nelanuthala S., Palam L.R., Pandey R., Mali R.S., Liu Y., Kelley M.R. (2018). Inhibition of Inflammatory Signaling in Tet2 Mutant Preleukemic Cells Mitigates Stress-Induced Abnormalities and Clonal Hematopoiesis. Cell Stem Cell.

[129] Sano S., Walsh K. (2021). Hematopoietic JAK2(V617F)-mediated clonal hematopoiesis: AIM2 understand mechanisms of atherogenesis. J. Cardiovasc. Aging.

[130] Sano S., Wang Y., Yura Y., Sano M., Oshima K., Yang Y., Katanasaka Y., Min K.D., Matsuura S., Ravid K. (2019). *JAK2^V617F^*-Mediated Clonal Hematopoiesis Accelerates Pathological Remodeling in Murine Heart Failure. JACC Basic Transl. Sci..

[131] Mead A. (2022). Inflammatory Crosstalk Drives Clonal Hematopoiesis. Hematologist.

[132] Kovtonyuk L.V., Caiado F., Garcia-Martin S., Manz E.M., Helbling P., Takizawa H., Boettcher S., Al-Shahrour F., Nombela-Arrieta C., Slack E. (2022). IL-1 mediates microbiome-induced inflammaging of hematopoietic stem cells in mice. Blood.

[133] Meisel M., Hinterleitner R., Pacis A., Chen L., Earley Z.M., Mayassi T., Pierre J.F., Ernest J.D., Galipeau H.J., Thuille N. (2018). Microbial signals drive pre-leukaemic myeloproliferation in a Tet2-deficient host. Nature.

[134] Lee Y.S., Kim T.Y., Kim Y., Kim S., Lee S.H., Seo S.U., Zhou B.O., Eunju O., Kim K.S., Kweon M.N. (2021). Microbiota-derived lactate promotes hematopoiesis and erythropoiesis by inducing stem cell factor production from leptin receptor+ niche cells. Exp. Mol. Med..

[135] Liu L., Shah K. (2022). The Potential of the Gut Microbiome to Reshape the Cancer Therapy Paradigm: A Review. JAMA Oncol..

[136] Woerner J., Huang Y., Hutter S., Gurnari C., Sanchez J.M.H., Wang J., Huang Y., Schnabel D., Aaby M., Xu W. (2022). Circulating microbial content in myeloid malignancy patients is associated with disease subtypes and patient outcomes. Nat. Commun..

[137] Zhang C.R.C., Nix D., Gregory M., Ciorba M.A., Ostrander E.L., Newberry R.D., Spencer D.H., Challen G.A. (2019). Inflammatory cytokines promote clonal hematopoiesis with specific mutations in ulcerative colitis patients. Exp. Hematol..

[138] Arends C.M., Weiss M., Christen F., Eulenberg-Gustavus C., Rousselle A., Kettritz R., Eckardt K.U., Chan W., Hoyer K., Frick M. (2020). Clonal hematopoiesis in patients with anti-neutrophil cytoplasmic antibody-associated vasculitis. Haematologica.

[139] Dharan N.J., Yeh P., Bloch M., Yeung M.M., Baker D., Guinto J., Roth N., Ftouni S., Ognenovska K., Smith D. (2021). HIV is associated with an increased risk of age-related clonal hematopoiesis among older adults. Nat. Med..

[140] Ertz-Archambault N., Kosiorek H., Taylor G.E., Kelemen K., Dueck A., Castro J., Marino R., Gauthier S., Finn L., Sproat L.Z. (2017). Association of Therapy for Autoimmune Disease with Myelodysplastic Syndromes and Acute Myeloid Leukemia. JAMA Oncol..

[141] Kusne Y., Fernandez J., Patnaik M.M. (2021). Clonal hematopoiesis and VEXAS syndrome: Survival of the fittest clones?. Semin. Hematol..

[142] Bhattacharya R., Zekavat S.M., Pirruccello J., Griffin G.K., Bick A.G., Natarajan P. (2020). Abstract 16686: Improved Diet Quality is Associated with Lower Prevalence of Clonal Hematopoiesis of Indeterminate Potential. Circulation.

[143] Haring B., Reiner A.P., Liu J., Tobias D.K., Whitsel E., Berger J.S., Desai P., Wassertheil-Smoller S., LaMonte M.J., Hayden K.M. (2021). Healthy Lifestyle and Clonal Hematopoiesis of Indeterminate Potential: Results from the Women’s Health Initiative. J. Am. Heart Assoc..

[144] Miller P.G., Qiao D., Rojas-Quintero J., Honigberg M.C., Sperling A.S., Gibson C.J., Bick A.G., Niroula A., McConkey M.E., Sandoval B. (2022). Association of clonal hematopoiesis with chronic obstructive pulmonary disease. Blood.

[145] Pasupuleti S.K., Ramdas B., Burns S.S., Kumar R., Pandhiri T.R., Sandusky G., Yu Z., Honigberg M., Bick A.G., Griffin G.K. (2021). Obesity-Induced Inflammation Co-Operates with Clonal Hematopoiesis of Indeterminate Potential (CHIP) Mutants to Promote Leukemia Development and Cardiovascular Disease. Blood.

[146] Szabo G., Saha B. (2015). Alcohol’s Effect on Host Defense. Alcohol. Res..

[147] Calder P.C., Ahluwalia N., Brouns F., Buetler T., Clement K., Cunningham K., Esposito K., Jonsson L.S., Kolb H., Lansink M. (2011). Dietary factors and low-grade inflammation in relation to overweight and obesity. Br. J. Nutr..

[148] Bullinger L., Dohner K., Dohner H. (2017). Genomics of Acute Myeloid Leukemia Diagnosis and Pathways. J. Clin. Oncol..

[149] Warren J.T., Link D.C. (2020). Clonal hematopoiesis and risk for hematologic malignancy. Blood.

[150] Steensma D.P. (2019). The Clinical Challenge of Idiopathic Cytopenias of Undetermined Significance (ICUS) and Clonal Cytopenias of Undetermined Significance (CCUS). Curr. Hematol. Malig. Rep..

[151] Malcovati L., Galli A., Travaglino E., Ambaglio I., Rizzo E., Molteni E., Elena C., Ferretti V.V., Catricala S., Bono E. (2017). Clinical significance of somatic mutation in unexplained blood cytopenia. Blood.

[152] Jan M., Snyder T.M., Corces-Zimmerman M.R., Vyas P., Weissman I.L., Quake S.R., Majeti R. (2012). Clonal evolution of preleukemic hematopoietic stem cells precedes human acute myeloid leukemia. Sci. Transl. Med..

[153] Shlush L.I., Zandi S., Mitchell A., Chen W.C., Brandwein J.M., Gupta V., Kennedy J.A., Schimmer A.D., Schuh A.C., Yee K.W. (2014). Identification of pre-leukaemic haematopoietic stem cells in acute leukaemia. Nature.

[154] Gibson C.J., Lindsley R.C., Tchekmedyian V., Mar B.G., Shi J., Jaiswal S., Bosworth A., Francisco L., He J., Bansal A. (2017). Clonal Hematopoiesis Associated with Adverse Outcomes after Autologous Stem-Cell Transplantation for Lymphoma. J. Clin. Oncol..

[155] Gillis N.K., Ball M., Zhang Q., Ma Z., Zhao Y., Yoder S.J., Balasis M.E., Mesa T.E., Sallman D.A., Lancet J.E. (2017). Clonal haemopoiesis and therapy-related myeloid malignancies in elderly patients: A proof-of-concept, case-control study. Lancet Oncol..

[156] Takahashi K., Wang F., Kantarjian H., Doss D., Khanna K., Thompson E., Zhao L., Patel K., Neelapu S., Gumbs C. (2017). Preleukaemic clonal haemopoiesis and risk of therapy-related myeloid neoplasms: A case-control study. Lancet Oncol..

[157] Steensma D.P., Bolton K.L. (2020). What to tell your patient with clonal hematopoiesis and why: Insights from 2 specialized clinics. Blood.

